# Supplementary material for: New Sulfur Organic Polymer-Concrete Composites Containing Waste Materials: Mechanical Characteristics and Resistance to Biocorrosion
Source: Materials (Basel). 2019 Aug 15;12(16):2602. doi: 10.3390/ma12162602 (PMC6721233; doi:10.3390/ma12162602)
Supplement: Supplementary file 1 [file materials-12-02602-s001.pdf]

materials

MDPI

Article

New Sulfur Organic Polymer-Concrete Composites Containing Waste Materials: Mechanical Characteristics and Resistance to Biocorrosion

Beata Gutarowska [1], Renata Kotynia [2], Dariusz Bieliński [3], Rafał Anyszka [3], Jakub Wręczycki [3], Małgorzata Piotrowska [1], Anna Koziróg [1], Joanna Berłowska [1,]* and Piotr Dziugan [1,]

1 Institute of Technology Fermentation and Microbiology, Faculty of Biotechnology and Food Science, Lodz University of Technology, Wolczanska 171/173, 90-924 Lodz, Poland
2 Department of Concrete Structures, Faculty of Civil Engineering, Architecture and Environmental Engineering, Lodz University of Technology, Al. Politechniki 6, 90-924 Lodz, Poland
3 Institute of Polymer and Dye Technology, Faculty of Chemistry, Lodz University of Technology, Stefanowskiego 12/16, 90-924 Lodz, Poland
* Correspondence: joanna.berlowska@p.lodz.pl

Received: 15 July 2019; Accepted: 12 August 2019; Published: 15 August 2019

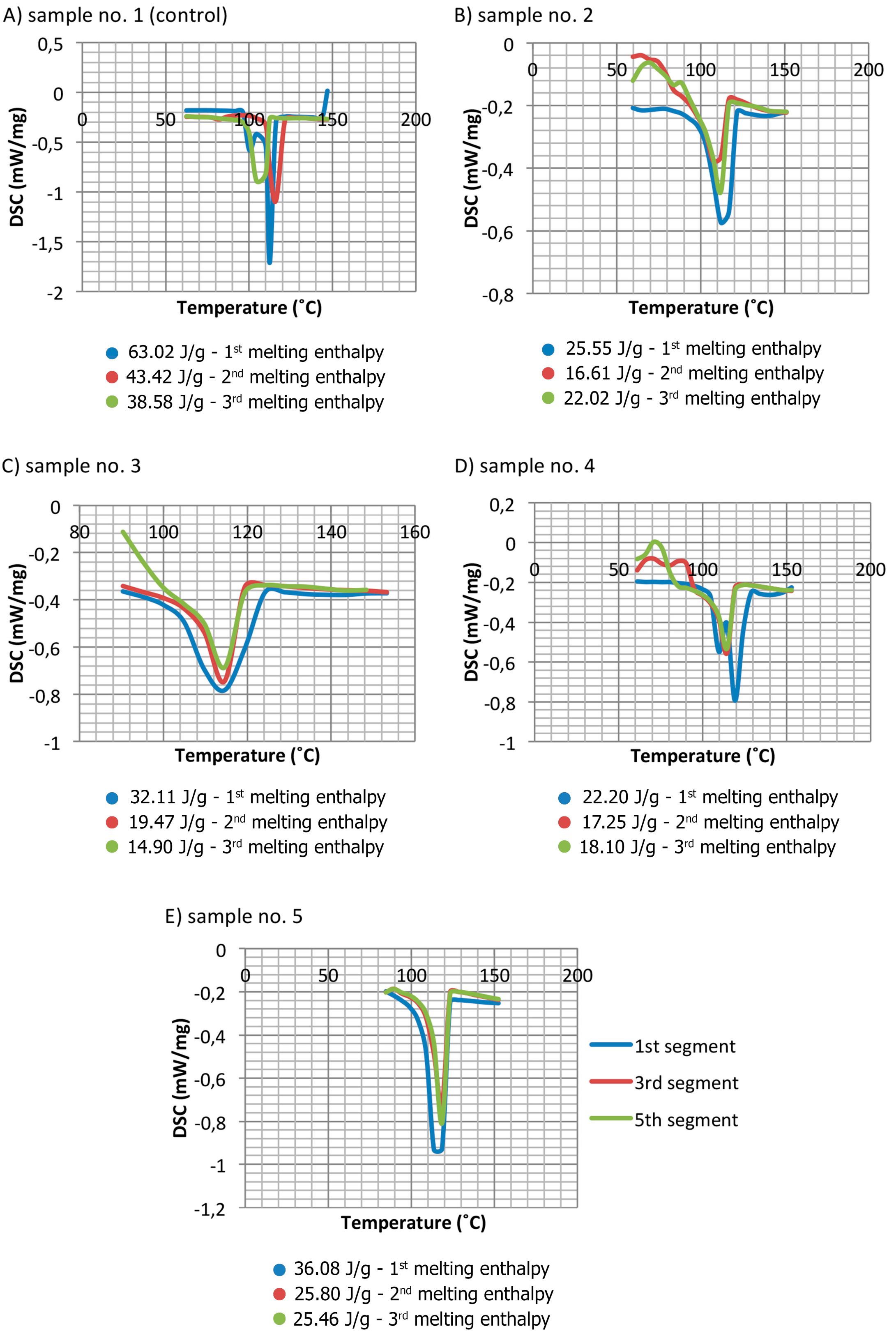

Figure S1. DSC curves for melting point following repeated melting of copolymers.

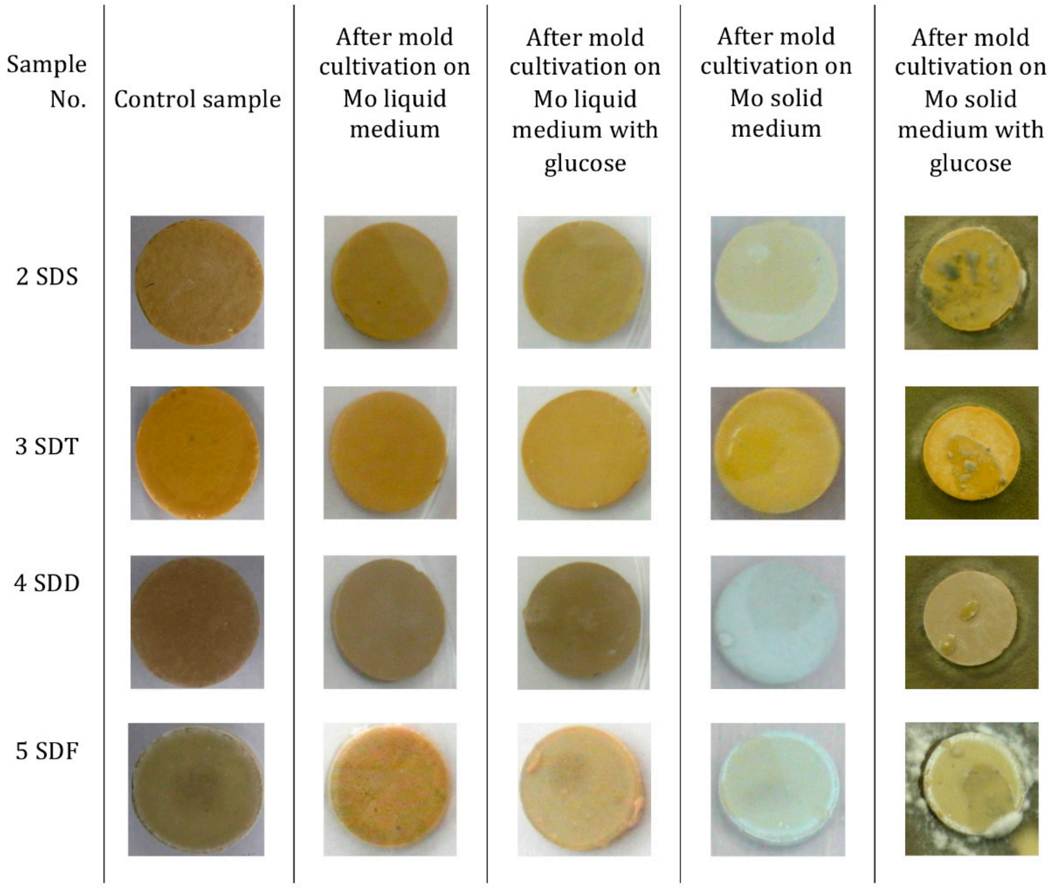

Figure S2. Sulfur copolymers after mold cultivation on liquid and solid Mo media with and without the addition of glucose.

Table S1. Dry mass of mycelium after incubation of molds on liquid Mo medium containing polymers with and without the addition of glucose.

Sample No.	Dry mass of mycelium [g/sample]	
	After mold cultivation on Mo liquid medium	After mold cultivation on Mo liquid medium with glucose
Control Mo	X: 0.004[a]	X: 0.242[a]
	SD: 0.002	SD: 0.076
2 SDS	X: 0.007[a]	X: 0.259[a]
	SD: 0.001	SD: 0.098
3 SDT	X: 0.018 [a]	X: 0.303 [a]
	SD: 0.015	SD: 0.038
4 SDD	X: 0.009 [a]	X: 0.162 [a]
	SD: 0.001	SD: 0.068
5 SDF	X: 0.018 [a]	X: 0.207 [a]
	SD: 0.017	SD: 0.089

*control medium without polymers # sample (discs with a diameter of 20 mm and thickness of 2mm) on liquid medium, volume 250 ml.

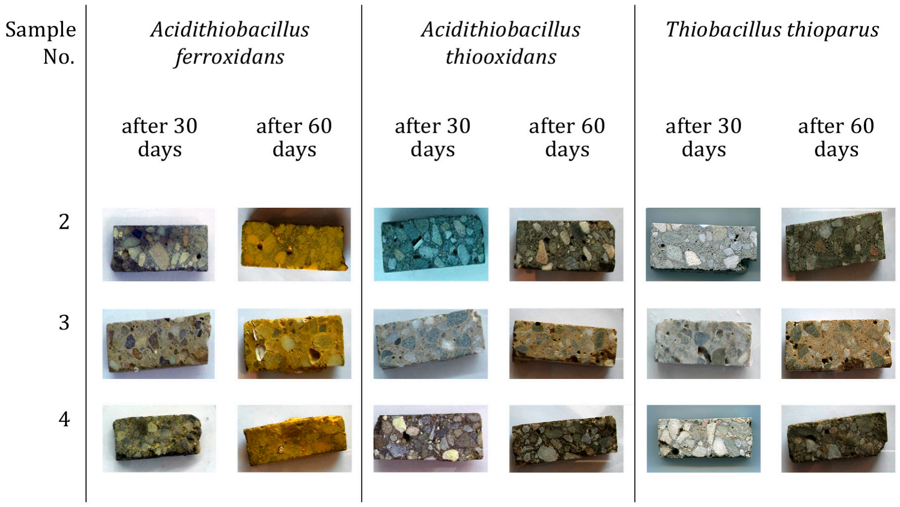

Figure S3. Macroscopic observations of sulfur concrete samples after incubation with bacteria.

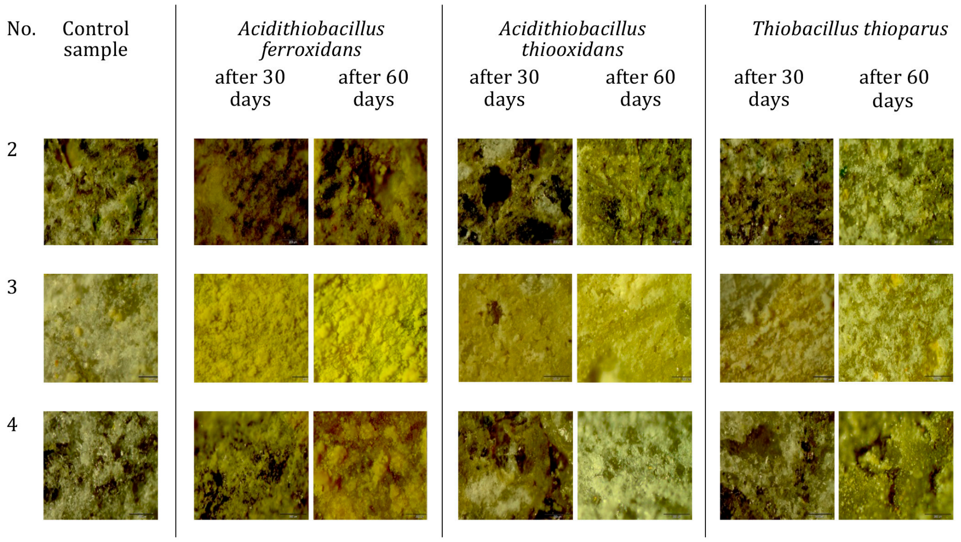

Figure S4. Microscopic observation of sulfur-concrete samples after incubation with bacteria.
